# Rare non-neoplastic gastrointestinal diseases–drugs and bugs

**DOI:** 10.1007/s00292-025-01501-2

**Published:** 2025-11-24

**Authors:** Lena Angerer, Tobias Zauner, Rupert Langer

**Affiliations:** Department of Clinical Pathology and Molecular Pathology, Kepler University Hospital and Johannes Kepler University, Krankenhausstr. 9, 4021 Linz, Austria

**Keywords:** Iatrogenic, Gastrointestinal, Medication, Histopathology, Parasites, Iatrogen, Gastrointestinal, Medikamente, Histopathologie, Parasiten

## Abstract

Rare diseases of the gastrointestinal (GI) tract present a significant diagnostic challenge. This paper focusses on two particular groups of conditions: drug-induced injuries and parasitic infections. With aging populations and increased use of complex pharmacologic regimens—including immunosuppressants and biologics—drug-related GI pathology is nowadays more frequently encountered. However, the histologic changes are often non-specific and overlap with more common conditions such as inflammatory bowel disease (IBD), ischemia, autoimmune diseases, or infections. Key patterns include lymphocytic or neutrophilic inflammation, crypt apoptosis, crypt abscesses, and architectural distortion. Certain agents, such as mycophenolate, olmesartan, immune checkpoint inhibitors, or ion exchange resins (e.g. sodium polystyrene sulfonate/kayexalate, sevelamer), have distinct but subtle histopathologic signatures. Parasitic infections, although less frequent in high-income countries, remain relevant due to global travel and migration. Organisms such as *Schistosoma, Strongyloides*, or *Giardia* can mimic IBD, neoplasia, or cause unexpected eosinophilic or granulomatous inflammation. Parasite ova may require special stains and careful morphologic assessment to be identified. Importantly, some helminths have been associated with chronic complications including cancer or fibrosis, thus underscoring the need for accurate recognition. For the practicing pathologist, these rare but impactful conditions demand a high index of suspicion, especially in cases with atypical histology or poor clinical correlation. Using some illustrative cases, this paper highlights diagnostic strategies and key morphologic features to improve recognition and avoid misdiagnosis of these underappreciated entities.

## Abstract (de)

Seltene Erkrankungen des Gastrointestinal(GI)-Trakts stellen oftmals eine diagnostische Herausforderung dar. Der Fokus der vorliegenden Arbeit liegt auf 2 speziellen Gruppen von deratigen Erkrankungsgruppen: medikamenteninduzierte Läsionen und parasitäre Infektionen. Medikamenteninduzierte Veränderungen treten insbesondere bei der zunehmend alternden Bevölkerung und dem erhöhtem Einsatz komplexer pharmakologischer Therapieregimes – einschließlich Immunsuppressiva und Biologika – heutzutage häufiger auf. Allerdings sind die histologischen Veränderungen oft unspezifisch und weisen überlappende Symptome mit häufigeren Erkrankungen wie chronisch-entzündlichen Darmerkrankungen (CED), Ischämie, Autoimmunerkrankungen oder Infektionen auf. Zu den wesentlichen Mustern gehören lymphozytäre oder neutrophile Entzündungen, Apoptosen, Kryptenabszesse und eine Störung der Schleimhautarchitektur. Bestimmte Substanzen, wie Mycophenolat, Olmesartan, Immuncheckpointinhibitoren oder Ionenaustauscherharze (z. B. Natriumpolystyrolsulfonat/Kayexalat, Sevelamer) weisen eindeutige, aber teils subtile histopathologische Merkmale auf. Parasitäre Infektionen sind zwar in Ländern mit hohem Einkommen weniger häufig, sind aber aufgrund z.B. weltweiter Reiseaktivitäten oder Migration relevante Diagnosen bzw. Differenzialdiagnosen. Organismen wie *Schistosoma*, *Strongyloides *oder *Giardia* können CED oder Neoplasien imitieren oder unerwartete eosinophile oder granulomatöse Entzündungen verursachen. Für die Identifizierung von Parasiteneiern werden möglicherweise spezielle Färbungen und eine sorgfältige morphologische Untersuchung benötigt. Einige Helmintheninfektionen gehen mit chronischen Komplikationen einschließlich Vernarbungen oder auch Tumoren einher, was die Notwendigkeit einer genauen Untersuchung unterstreicht. Vom praktisch tätigen Pathologen verlangen diese seltenen, aber gravierenden Erkrankungen ein hohes Maß an Umsicht, insbesondere in Fällen mit atypischer Histologie oder fehlender klinischer Korrelation. Anhand einiger anschaulicher Fälle werden in der vorliegenden Arbeit diagnostische Strategien und wesentliche morphologische Kennzeichen dargestellt, um die Erkennung dieser seltenen und teils unterschätzten Entitäten zu verbessern und Fehldiagnosen zu verhindern.

## Introduction

The gastrointestinal (GI) tract represents a dynamic interface between the host and a vast array of exogenous agents, both microbial and pharmacologic. Pathologists may encounter gastrointestinal pathology resulting from “bugs”—parasitic and microbial infections—as well as “drugs”—medication-induced injuries or adverse effects [1–4]. These processes may mimic each other clinically or histologically, often presenting with non-specific patterns of mucosal injury, inflammation, or architectural alteration [5, 6]. As travel and migration on the one hand and therapeutic complexity on the other increase, the spectrum of encountered pathogens and drug-related changes continues to broaden, but both conditions still are relatively uncommon in daily routine practice, making it all the more important to recognize and understand their key features when these rare cases do arise. In addition, the clinical presentation, however, is often not clear and can even be misleading, or clinical information are not available or stated.

This article presents some illustrative examples of infectious and drug-associated pathologies, highlighting diagnostic challenges and characteristic histologic features relevant to daily practice in gastrointestinal pathology.

## Drugs: medication-induced gastrointestinal pathology

Drug-induced gastrointestinal pathology is common yet often underrecognized—even though up to 8% of patients taking medications exhibit gastrointestinal side effects. Clinically, these side effects manifest in limited ways, including diarrhea, abdominal pain, constipation, nausea, vomiting, hemorrhage, or ulceration, reflecting the constrained repertoire of mucosal response patterns. Drugs may cause GI damage directly or via metabolites, excipients, or host reactions. Histopathologically, this overlap of injury patterns presents diagnostic challenges, as drug-induced changes may mimic primary gastrointestinal diseases. Limited clinical information and a lack of specific features often hinder definitive diagnosis, thus underscoring the need for heightened suspicion in ambiguous cases [7, 8].

Particular attention should be given when observing:

- unusual inflammatory patterns, especially with prominent lymphocytic or eosinophilic infiltration.
- marked nuclear pleomorphism and cytologic atypia with preserved architecture.
- increased apoptosis and mitoses.
- dilated, atrophic crypts or glands.
- ischemic-type lesions in patients without vascular disease.

Well-known risk scenarios include chronic renal insufficiency, hypertension, autoimmune diseases, transplantation, malignancy, and polypharmacy, especially in elderly patients. However, abuse of medications can also occur in young populations, including lifestyle drugs or painkillers [8].

**Fig. 1:**
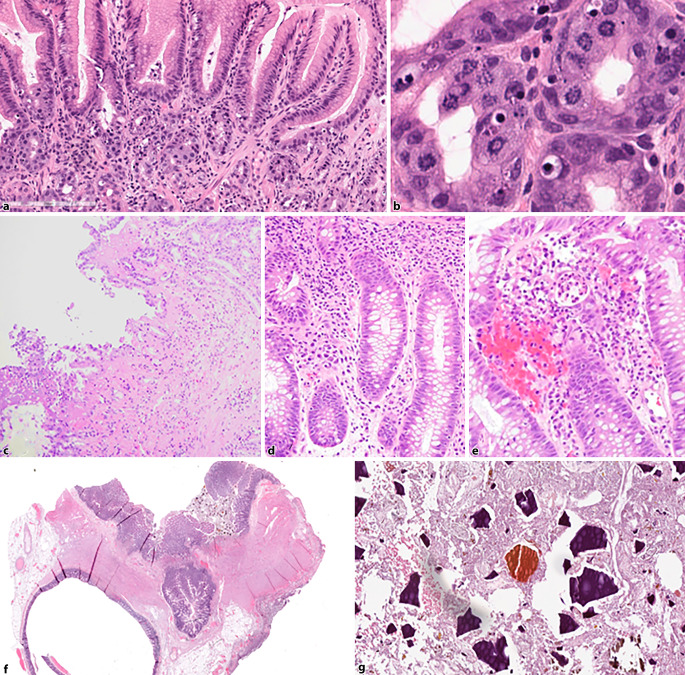
Examples of drug-induced pathologic changes in the gastrointestinal tract: **a,** **b** colchicine-induced gastropathy with ring mitoses and apoptosis (**a** overview, **b** magnification); **c** ischemic changes in the stomach caused by a nonsteroidal anti-inflammatory drug; **d,** **e** immune checkpoint inhibitor-induced colitis with increased intraepithelial lymphocytes and apoptosis (**d**) or active colitis (**e**); **f,** **g** Kayexalate (blue-purple) and sevelamer (yellowish-red) crystals in a sigmoid resection specimen with diverticulosis and acute damage of adjacent mucosa due to the crystals (**f** overview, **g** magnification)

Table 1 lists frequently observed patterns and respective drugs causing medication related gastrointestinal damages. In the following text, some examples of drug-induced gastrointestinal pathologic conditions are presented (see also Fig. 1).

### NSAID-induced gastrointestinal injury

The clinical presentation of nonsteroidal anti-inflammatory drug (NSAID)-induced injury is often nonspecific, ranging from iron-deficiency anemia due to occult blood loss to symptoms of subacute small bowel obstruction in cases of chronic stricture formation, also known as “diaphragm disease.” A critical clinical feature is the poor correlation between upper GI symptoms and the presence of significant mucosal damage. Severe life-threatening complications like bleeding or perforation can occur without any prior warning symptoms. While NSAIDs can damage the entire gastrointestinal tract, the histopathologic findings vary by location. Due to the formulation of most modern medications, including NSAIDs, leading to release of the active components in the stomach or further along the intestinal tract, NSAID-induced damage in the esophagus is rare. In the stomach—the primary site of NSAID-induced damage—inhibition of prostaglandin synthesis by inhibition of cyclo-oxygenase is the driving force behind NSAID-induced injury. While NSAIDs classically cause ulceration of the stomach mucosa due to the missing protective effect of prostaglandins, a reactive gastropathy defined by foveolar hyperplasia with a characteristic corkscrew appearance, mucosal edema, and fibromuscular hyperplasia of the lamina propria, all with a notable paucity of inflammatory cells, may also be the only NSAID-associated damage present [8].

In the small intestine, NSAIDs mostly cause unspecific ulceration and erosion; however, a rare but significant complication of long-term NSAID use is diaphragm disease, characterized by thin, concentric septa that cause stenosis. Histologically, these diaphragms consist of a core of submucosal fibrosis admixed with disorganized smooth muscle, unmyelinated nerve bundles, and angiomatous blood vessels. In the large intestine, NSAID-associated injury typically manifests as nonspecific, patchy inflammation, in some cases with erosion and ulceration, predominantly in the right colon. The inflammatory infiltrate is variable and can be mixed lymphoplasmacytic and neutrophilic, predominantly neutrophilic (mimicking infectious colitis), or predominantly lymphocytic. While mild crypt architectural disarray is common, features of established chronicity like significant crypt distortion (branching, atrophy) or prominent basal plasmacytosis can be absent [9, 10].

The differential diagnosis of NSAID-induced enterocolitis primarily includes Crohn’s disease (CD), ischemic colitis, and infectious colitis. Unlike CD, NSAID injury lacks granulomas, transmural inflammation, and deep fissuring ulcers. Histologically, NSAID-induced inflammation is typically less severe, with a milder lymphoplasmacytic infiltrate and less architectural distortion than in active CD. NSAIDs can also induce an ischemic-type colitis, although the inflammation in NSAID colitis seems to be generally milder than that seen in primary ischemic colitis. The acute neutrophilic pattern can be histologically indistinguishable from infectious colitis, making the exclusion of pathogens via clinical and microbiologic studies essential for diagnosis [10, 11].

### Colchicine- and taxane-induced gastrointestinal injury

Colchicine and taxanes interfere with microtubule function, leading to mitotic arrest [12, 13]. Clinically, this manifests as nausea, vomiting, abdominal pain, and diarrhea for both drug classes. For patients taking colchicine, these gastrointestinal symptoms are the earliest and most frequent manifestations of toxicity, which can be severe and even fatal, particularly in those with renal or hepatic impairment. For patients on taxane-based chemotherapy (e.g., docetaxel or paclitaxel), similar GI side effects are common but are generally considered part of the expected cytotoxic effects of the treatment. The histopathologic effects are strikingly similar for both drug classes and are characteristically confined to the proliferative compartments of the epithelium, such as the crypts, while sparing the mature, differentiated surface epithelium. The hallmark histologic features include a marked increase in apoptosis within the crypts and accumulation of epithelial cells arrested in metaphase. Many of these arrested cells display a pathognomonic “ring mitosis,” where condensed chromosomes form a circular or wreath-like configuration. These changes can be accompanied by reactive epithelial atypia, including nuclear enlargement, pseudo-stratification, and a loss of normal nuclear polarity. The differential diagnosis is unique because the histologic findings for colchicine and taxanes are identical. The distinction is entirely clinical and dictates management: for a patient on colchicine, these findings are diagnostic of toxicity and require immediate drug cessation; for a patient on taxane-based chemotherapy, the same findings represent the intended therapeutic effect and may be seen in asymptomatic patients, not necessarily indicating toxicity. The reactive atypia can also mimic high-grade dysplasia. The crucial distinguishing feature is that the drug effect is sharply limited to the crypt bases, with preserved epithelial maturation toward the surface—a feature that is absent in true dysplasia, which is defined by the extension of atypical features to the surface epithelium [13].

### Immune checkpoint inhibitor (ICI)-induced colitis

The primary clinical symptom of ICI-induced colitis is diarrhea, which is often accompanied by abdominal pain and fever. The onset is typically delayed, with a median time of 6–8 weeks after initiation of anti-CTLA‑4 therapy and 3–6 months for anti-PD-1/PD-L1 agents. The condition can progress rapidly to severe complications like toxic megacolon or perforation if not treated [14]. Histopathologically, ICI-induced colitis is a great mimicker, presenting with a spectrum of overlapping injury patterns; the presence of multiple patterns within the same biopsy set is a strong clue to the diagnosis. The most common patterns include the following: an acute active colitis with neutrophilic cryptitis and crypt abscesses, resembling infectious colitis; a chronic active pattern with architectural distortion and basal plasmacytosis, mimicking inflammatory bowel disease (IBD); an apoptotic pattern with prominent crypt cell apoptosis, resembling graft-versus-host disease (GVHD); and patterns that are histologically identical to lymphocytic or collagenous colitis [15]. Given this histologic diversity, ICI-induced colitis is fundamentally a diagnosis of exclusion. The most critical differential diagnosis is infectious colitis, which must be ruled out by microbiologic studies before initiating immunosuppressive therapy. When differentiating from an IBD flare, the presence of prominent crypt apoptosis and less pronounced basal plasmacytosis strongly favors ICI colitis, whereas features like true granulomas or deep fissuring ulcers are diagnostic of Crohn’s disease. Compared to GVHD, the apoptotic pattern of ICI colitis is a close mimic but is typically accompanied by a more robust inflammatory infiltrate, whereas classic acute GVHD is characterized by a paucicellular lamina propria. Finally, while it can be histologically identical to idiopathic microscopic colitis, the ICI-induced form often has a more aggressive clinical course and may show overlapping features like cryptitis or apoptosis not typically seen in the idiopathic setting [8, 16].

### Crystals (adsorbents and resins)

Adsorbents and therapeutic resins, such as sodium polystyrene sulfonate (Kayexalate; used to treat hyperkalemia by binding intraluminal potassium) and sevelamer (a phosphate binder for hyperphosphatemia in chronic kidney disease), are nonabsorbable medications frequently encountered microscopically in gastrointestinal specimens. At high concentrations, these agents may cause mucosal injury, including ischemia, necrosis, stromal fibrosis, ulceration, and erosion with crystalline deposition. This can be detectable throughout the GI tract—with crystals occasionally seen in luminal exudate [4].

Kayexalate is associated with ischemia, mucosal necrosis, and potential perforation, although it can also be present without overt injury. Histologically, Kayexalate crystals are bright purple with a distinctive fish-scale pattern in H&E staining. Sevelamer can similarly cause GI mucosal injury; its crystals are yellow or pink, also displaying a fish-scale appearance. Other tablet fragments, including bile acid sequestrants, generally do not lead to mucosal damage but may be identified microscopically as pill fragments in tissue sections. Recognition of these characteristic crystalline or pill-residue findings is critical for accurate diagnosis and to avoid misinterpretation as primary pathologic processes [7, 16].

## Bugs: parasitic infections

Gastrointestinal parasitic infections remain a significant yet often underappreciated contributor to human disease worldwide. While most prevalent in tropical and subtropical regions, migration, travel, and globalization have increased their recognition in temperate countries, presenting challenges for both clinicians and pathologists. The spectrum of parasites affecting the gastrointestinal tract is broad, ranging from protozoa such as *Entamoeba histolytica* and *Giardia lamblia* to helminths including *Ascaris lumbricoides, Strongyloides stercoralis*, and cestodes. Their manifestations may vary from asymptomatic colonization to severe, life-threatening disease, often with nonspecific clinical and endoscopic findings [2].

**Fig. 2:**
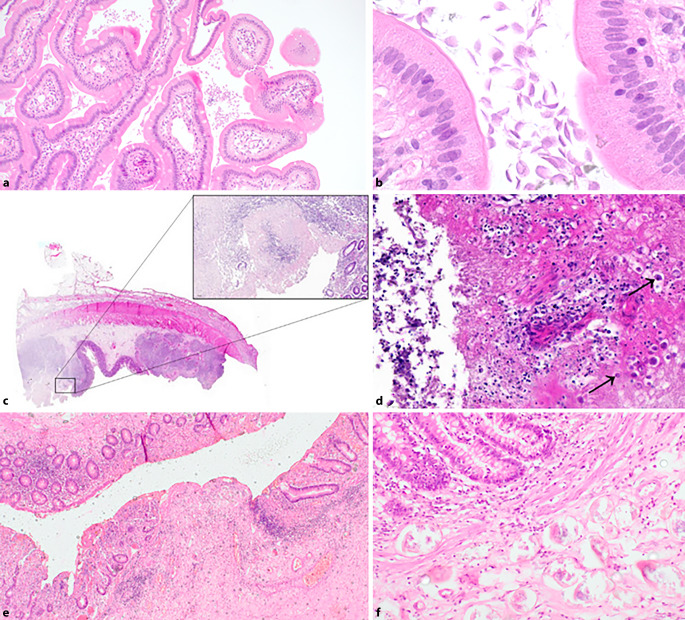
Examples of parasitic infections in the gastrointestinal tract: **a,** **b** lambliasis in the duodenum with considerable amounts of *Giardia lamblia* in the intervillous space (**a** overview, **b** magnification); **c,** **d** ulceration in the intestine due to *Entaboeba histolyticum* infection (**c** overview, **d** magnification; from [20]); **e,** **f** schistosomiasis in the colon with ulcerations and lots of partially calcified eggs, here without significant granulomatous reaction (**e** overview, **f** magnification)

Clinically, parasitic infections and associated inflammatory changes may mimic neoplasia or chronic inflammatory disorders. The range of histopathologic changes reflects the complex interactions of the parasites with the host tissue. They can cause mucosal damage, inflammation, and alterations in the epithelial architecture, which are critical for understanding disease progression and host response. For pathologists, awareness of the morphologic spectrum of parasites and their common diagnostic pitfalls remains highly relevant in daily practice. In the following, some examples are presented, including their clinical and pathologic characteristics (see also Fig. 2).

### Lambliasis

*Giardia lamblia* (syn. *G. duodenalis, G. intestinalis*) is a protozoan parasite causing an intestinal infection called giardiasis. It is one of the most common causes of waterborne nonviral and nonbacterial diarrheal disease, with over 300 million cases reported worldwide each year. In 2022 there were more than 10,000 confirmed cases of giardiasis in the European Union. The infection can be asymptomatic or associated with diarrhea, abdominal pain, malabsorption, and weight loss. There are high-risk groups including immunocompromised persons, infants, children, travelers visiting highly endemic regions, and individuals who practice unprotected oral/anal sex [17].

*Giardia lamblia* undergoes two primary stages in its lifecycle: the infectious cyst stage and the proliferative trophozoite stage. The infectious cyst is transmitted through the fecal–oral route, mainly by ingesting contaminated water or food containing cysts of *Giardia*. Transmission from person to person is also common, whereas animal-to-person transmission is less frequent.

Once the cysts reach the duodenum, they undergo excystation, releasing the trophozoites as the active form. This is stimulated by gastric acid and exposure to bile and pancreatic proteases. Symptoms occur during the trophozoite stage. The infection is mainly focused in the proximal small intestine. The trophozoites adhere to the microvilli of the enterocytes but remain noninvasive. Once dislodged, trophozoites pass through the small intestine and encyst upon reaching the colon. Excretion of cysts occurs through the feces.

*Giardia* trophozoites are pear-shaped and contain two nuclei. In profile they appear elliptical, resembling a “nail clipping,” and range in size from 10 to 20 μm in length. The infection causes villous atrophy and crypt hyperplasia in the small intestine. There is an increase in intraepithelial lymphocytosis and mild inflammation in the lamina propria. In most cases, however, the mucosa appears morphologically normal. Evaluation of the intervillous space is therefore a very important last look not to be missed by the pathologist when signing out duodenal biopsies [18].

### Amebiasis

*Entamoeba histolytica* is a protozoan parasite causing amebiasis. While 90% of *E. histolytica* infections remain asymptomatic, nearly 50 million people develop symptoms annually, resulting in up to 100,000 deaths each year. Infection is distributed worldwide, predominantly affecting areas with lower socioeconomic status and limited public health resources. Humans serve as the sole natural host. Transmission occurs primarily through fecal–oral contact via ingestion of food or water contaminated with cysts excreted in feces, while transmission through anal intercourse has also been documented.

*Entamoeba histolytica* exists in two life stages: the infectious cyst form and the ameboid trophozoite form. The cysts withstand gastric acidity, transit through the small intestine, and undergo excystation in the terminal ileum or colon to release the trophozoite stage. Trophozoites ingest bacteria and particulate matter, replicate by binary fission, and encyst within the colon. The cysts are shed into the environment through fecal excretion.

The infection is typically located in the cecum and the rectum. Although most cases remain asymptomatic, amoebic colitis typically manifests with a subacute, gradual onset of watery or bloody diarrhea, loss of appetite, abdominal pain, and weight loss. In rare instances patients may progress to fulminant colitis, presenting with fever, severe hemorrhagic diarrhea, and clinical signs of peritonitis. This critical condition is associated with intestinal necrosis, perforation, and toxic megacolon and is linked to a high mortality rate. Liver abscess represents the most frequent extraintestinal manifestation and may develop months to years following exposure in endemic regions. In the gastrointestinal tract, the infection may mimic other gastrointestinal disorders, including inflammatory bowel disease, ischemic colitis, and various bacterial infections such as those caused by *Clostridioides difficile, Shigella, Escherichia coli, Campylobacter*, and *Salmonella* [1, 2].

The most frequent findings are flask-shaped ulcers or erosions in the lamina propria of mucosa. A limited breach of the mucosa is found accompanied by lateral extension into the submucosal layer. The mucosal surface is covered with inflammatory exudate composed of inflammatory cells, necrotic debris, and fibrin. Trophozoites are identified within this exudate. Edema and goblet cell depletion are also seen in the mucosal layer. Trophozoites display round to ovoid morphology, ranging from 6 to 40 μm in size. They exhibit pseudo-podial projections, small round nuclei, and a peripheral rim of condensed chromatin. The cytoplasm stains positive with periodic acid–Schiff (PAS) and demonstrates a granular appearance. They also may be difficult to distinguish from histiocytes, a problem which can be solved by immunohistochemical stains against histiocytic markers, e.g., CD68 [19, 20].

### Schistosomiasis

*Schistosoma* is a genus of trematode parasites responsible for chronic schistosomiasis, a disease predominantly affecting the intestinal tract and hepatosplenic system. The infection involves multiple other organ systems, including the spleen, central nervous system, and urogenital tract. Six primary species of *Schistosoma* are known to infect humans: *Schistosoma japonicum, Schistosoma mansoni, Schistosoma haematobium, Schistosoma mekongi, Schistosoma interdental, *and *Schistosoma malayi* [21]. The International Agency for Research on Cancer (IARC) classifies *Schistosoma japonicum* infection as possibly carcinogenic to humans due to its association with liver cancer, while the link between *Schistosoma haematobium* infection and bladder cancer is well established and recognized as carcinogenic.

Intestinal schistosomiasis refers to the acute and chronic inflammation of the intestines caused by *Schistosoma* infection, particularly by *Schistosoma japonicum, Schistosoma mansoni, or Schistosoma mekongi*, but typically not *Schistosoma haematobium*. Transmission occurs upon contact with freshwater containing snails which are the intermediate hosts. Cercariae, the infectious form of the parasite, penetrates human skin. The eggs subsequently migrate to the hepatic venules, where they mature into adult worms. Adult worms then migrate to the mesenteric veins of the intestines or the vesical venous plexus, where females deposit eggs that are ultimately excreted in the feces or urine.

Gastrointestinal schistosomiasis commonly affects the descending colon, sigmoid colon, and rectum. Most cases remain asymptomatic, while other cases manifest with fever, diarrhea, abdominal pain, bowel obstruction, and hematochezia. Clinically, it can resemble other gastrointestinal conditions, such as Crohn’s disease, acute viral and bacterial infections, and other parasitic helminth infections. Mass-forming inflammatory reactions may resemble polyps or cancer. Cases with severe pneumatosis have also been reported [22]. The infection is linked to significant morbidity and mortality, especially among populations with inadequate sanitation and limited access to safe drinking water.

Macroscopically, intestinal schistosomiasis typically presents with yellow, granular mucosa, and may also manifest as polypoid lesions. Ulcerations, edema, and hemorrhages can be observed. Histopathologic findings include submucosal fibrosis, acute and chronic inflammation, multinucleated giant cells containing *Schistosoma* eggs, and non-necrotizing granulomas. Of note, *Schistosoma* eggs are PAS negative but may react differently to Ziehl–Neelsen (ZN) staining (i.e., *S. mansoni* eggs are ZN negative and *S. haematobium* are ZN positive). During disease progression, eggs undergo calcification, which is accompanied by a progressive accumulation of fibrotic tissue in the affected areas [23].

## Summary

This article highlights the diverse range of pathologic changes in the gastrointestinal tract caused by both infective agents, particularly parasites, as well as by medications. Both can each induce a spectrum of mucosal alterations, ulcers, inflammation, and other lesions—often with overlapping clinical and histological patterns. Accurate diagnosis relies on both thorough histopathologic assessment and awareness of patient history, given that infections and drug-induced injuries may mimic primary GI diseases or each other. For medications, characteristic findings—such as distinctive crystals like Kayexalate or sevelamer, or typical features of specific drugs such as ring mitoses observed under taxane/colchicine therapy—serve as important diagnostic clues but are not universally present. Detection of parasites, in turn, may be easier when obvious or abundant, but the clinical appearance may even mimic malignancies in case of large ulcerating inflammatory lesions. Close collaboration between pathologists and clinicians and consideration of travel, epidemiologic, and medication histories, for example, are essential for effective identification and management of these mimicking entities.

**Table 1:** Patterns of drug-induced morphologic changes in the gastrointestinal tract

Pattern	Localization	Medications (examples)
Chronic inflammation	All	NSAIDs, biologics, MMF, TNF inhibitors
Focal inflammation	All	NSAIDs, biologicals
Excessive apoptosis	All	NSAIDs, biologicals, TNF inhibitors, MMF, taxanes, colchicine, laxatives
Erosions and ulcerations	All	NSAIDs, Kayexalate
Mitoses	All	Taxanes, colchicine
Ischemic pattern	All	Digitalis, diuretics, cocaine, dopamine, NSAIDs, and many others
Autoimmune-like pattern	All	Biologicals, checkpoint inhibitors
Dilated glands or crypts	All	MMF, laxatives, chemotherapeutics
Malakoplakia	All	Steroids
Crystals	All	Kayexalate, sevelamer, cholestyramine
Eosinophils	All	NSAIDs, aspirin, biologics
Stenoses/strictures/diaphragm-like	All	NSAIDs
Villous atrophy	Small intestine	Sartans, MMF, biologicals, NSAIDs
Pseudomembranous colitis	Colon	Antibiotics, PPIs
Diverticular perforation	Colon	Steroids
Microscopic colitis	Colon	NSAIDs, PPIs, statins, psychotropic drugs, antibiotics

*NSAIDs* non steroidal antiinflammatory drugs, *MMF* mycophenolate mofetil, *TNF* tumor necrosis factor, *PPI* protone pump inhibitor

## Declarations

For this article no studies with human participants or animals were performed by any of the authors. All studies mentioned were in accordance with the ethical standards indicated in each case.

The supplement containing this article is not sponsored by industry.
